# Update on Oral Appliance Therapy for OSA

**DOI:** 10.1007/s40675-017-0080-5

**Published:** 2017-07-10

**Authors:** M. Marklund

**Affiliations:** Department of Odontology, Medical Faculty, Umeå University, SE-906 87 Umeå, Sweden

**Keywords:** Oral appliances, Mandibular advancement devices, Mandibular repositioning appliances

## Abstract

**Purpose of Review:**

The majority of the adult population is affected by obstructive sleep apnea (OSA), according to recent epidemiological research. Oral appliance (OA) therapy is increasingly recommended, particularly for patients with milder OSA. This review updates the evidence in favor of OA therapy.

**Recent Findings:**

A high level of evidence shows that OA is effective in the treatment of OSA, but continuous positive airway pressure (CPAP) is more efficient. Higher adherence with OAs may compensate for this difference. Daytime sleepiness is better treated with CPAP than with OA in patients with severe OSA. In patients with milder OSA, it is unclear whether sleepiness is significantly reduced. The long-term effectiveness of OAs is uncertain because of side-effects and the risk of OSA deterioration.

**Summary:**

OAs are effective, but their efficacy is more variable than that of CPAP. More research is needed about the mechanism of action of OA, subjective effects and long-term health outcomes.

## Introduction

Obstructive sleep apnea (OSA) is highly prevalent in the adult population [1]. Eighty-four per cent of men and 61% of women aged 40 years or older have an apnea-hypopnea index (AHI) of ≥5. A slightly lower prevalence is found, if symptoms or comorbidities such as hypertension are included in the diagnosis [2]. The majority of the subjects have milder forms of the disease, particularly in the younger age groups [1, 2]. These high numbers of potential patients have initiated a discussion about treatment needs in those with milder forms of the disease [3]. Oral appliances (OAs) have become an increasingly common alternative for patients with OSA of varying severity, although mainly those with milder OSA. Consequently, there is a need for a continuous update of knowledge about the various aspects of this therapy. This review will summarize the present evidence relating to OA treatment and elucidate some areas that are as yet less well studied. Examples of these topics include the more exact indications, patient-oriented aspects of the therapy in terms of symptomatic effects, side-effects, the importance of device design, and the long-term health outcomes.

## Mechanism of Action

Oral appliances reposition the lower jaw forwards in order to increase the upper airway volume and reduce pharyngeal collapsibility [4]. The upper airway enlarges, particularly in its lateral dimension at velopharyngeal level, and the tongue is displaced anteriorly [4–6]. Various underlying pathophysiologies between OSA patients are differently affected by the mechanism of action of OAs. OSA patients may suffer, to varying degrees, from upper airway anatomical abnormalities, increased pharyngeal collapsibility, an overly sensitive ventilatory control system (high loop gain), and a reduced arousal threshold [7•]. OAs improve pharyngeal collapsibility, while loop gain, arousal threshold, and dilator muscular activity are unchanged [8••]. Patients with mild OSA are thought to be better suited to the mechanism of OAs than patients with more severe OSA. They generally have less collapsible airways [7•, 8••], and their pharynx will increase more, along the whole of its length, compared with patients with more severe disease [9].

In conclusion, the mechanism of action of OAs is less predictable than that of continuous positive airway pressure (CPAP), even in the longer term [10••, 11••, 12]. An OA can therefore be used as a sole treatment or in combination with other sleep apnea treatments such as CPAP or positional therapy, when the treatment is not totally successful in itself [13, 14, 15•]. On the other hand, an OA can help to reduce the side-effects of CPAP machines in patients who suffer from uncomfortably high pressures [16, 17].

## Short-Term Effects on Apneas and Hypopneas

The apnea-hypopnea index is effectively reduced by OA compared with placebo interventions [11••]. The nightly oxygenation is improved [10••, 11••]. Treatment with CPAP is more effective than OA treatment in reducing the AHI, according to all the studies comprising patients with mild to severe OSA [10••, 11••]. With CPAP, the nightly oxygenation is further restored compared with OA [10••].

## Predicting Success

In general, larger repositioning of the lower jaw forwards will have a better effect on the apnea-hypopnea index, although there is no linear relationship [18]. Differences in the ability to advance the lower jaw forwards between patients will therefore, to some extent, modify the potential to reduce or eliminate obstructive sleep apneas. Milder OSA patients have a greater chance of treatment success with OA therapy than patients with severe OSA. In this group of patients, the success rate is fairly equal between OA and CPAP [19, 20]. Severe OSA patients can be successfully treated with OA [19], but CPAP is more likely to eliminate obstructive sleep apneas and hypopneas in these patients [10••, 11••]. OAs can, however, be recommended as a second line treatment in CPAP-intolerant patients [21, 22].

A new approach to predicting treatment success with OAs highlights the variability in OSA pathophysiology between patients. Patients with less collapsible upper airways and a less sensitive ventilatory control system are more likely to benefit from OA therapy [7•, 8••]. Patients with milder OSA have, in general, a better passive upper airway anatomy and a less collapsible airway, but may differ in the presence of non-anatomic traits [7•]. These characteristics might therefore constitute useful additional negative predictors of success for OA therapy in this group of patients.

In line with these results, good prediction potential has been detected by the verified widening of the pharynx visualized by nasendoscopy [6, 23–25] or a sufficient reduction in AHI during an overnight test with a remotely controlled device [26•, 27].

Results from clinical routine such as a lower CPAP pressure [28, 29] or existing supine-position-dependent sleep apnea can also be useful predictors of success [30–34]. Some conflicting results about the value of supine dependency as a predictor of success can probably be explained by differences in the potential of various devices to stabilize the lower jaw in a forward position during sleep. The fixation of the lower jaw to the upper jaw to achieve the intended forward repositioning might be particularly important in the supine position [35].

Lower age or non-obesity at baseline or female sex have been related to success, although these predictors have variable strength and represent less useful predictors in clinical practice [20, 32, 36]. Weight increase during treatment has, however, been related to treatment failure [32]. Cephalometric evaluations of morphological variables are inconsistent predictors of success [37•]. Elderly people may be satisfactorily treated with OAs, although there are few studies in this area [38].

In conclusion, there is a high level of evidence of a satisfactory effect on sleep apnea by OAs. The variability in treatment response means, however, that CPAP will always represent a more efficient alternative. It is therefore important for the future to find validated predictors of a successful apnea-hypopnea reduction with OA treatment. Until more knowledge is available, snoring and mild to moderate OSA constitute the primary indications for OA treatment. A confirmed widening of the upper airway or AHI reduction during a prediction test can also be used, if the methodology is clinically available.

## Symptomatic Effects

The effect on daytime sleepiness from obstructive sleep apnea treatments is unclear, particularly in subjects within the mildest spectrum of disease severity. Many patients report less daytime sleepiness according to the Epworth Sleepiness Scale (ESS) score with OA treatment compared with not having a device in situ, but some of this may be related to placebo effects [39, 40•, 41]. In the nine randomized controlled studies [39, 40•, 41–47] that compare OA with an intraoral placebo device, only one cross-over study found a significant difference in the ESS score between OA and a placebo device [39]. None of the five parallel RCT studies reported a lower ESS score with OA compared with a placebo device [40•, 41–43, 45]. Two randomized controlled studies comprised milder OSA patients; i.e., those who are primarily indicated for this type of therapy. There was no effect on the ESS score compared with placebo intervention in these studies [40•, 44]. One of these two studies concentrated on symptomatic effects, and there were no effects on fatigue, prospective reports of daytime sleepiness measured by the Karolinska Sleepiness Scale, objective tests of sleepiness by the Osler test, or quality of life in that study [40•]. The summarized results in a recent meta-analysis report no effect on the ESS score from OAs compared with placebo in a group of patients with moderate sleep apnea [11••]. Nor was there any difference in the ESS score between CPAP and OAs in this group of patients [11••]. No placebo controlled studies of mild OSA patients had been published when this meta-analysis was performed. Consequently, it is uncertain, whether daytime sleepiness and quality of life are significantly reduced by any OSA treatment in patients with milder OSA [11••, 48]. In contrast, patients with more severe OSA experience a reduction in daytime sleepiness as a result of OA compared with a placebo intervention, but CPAP is more effective [11••]. Most likely, mild to moderate OSA causes less pronounced daytime sleepiness than previously assumed, while other conditions and diseases may explain daytime sleepiness. These recent findings also elucidate why as much as one third of patients who are treated with either CPAP or OA are still sleepy, defined as an ESS score of above 10, despite a successful sleep apnea reduction [49, 50•].

Regarding other symptoms that are related to untreated OSA, two studies have found a significant positive effect on symptoms of restless legs by OA treatment versus placebo interventions [40•, 51]. A number of other OSA-related symptoms, such as headaches, nasal congestion, and insomnia, were lower with active treatment than without the device in one of these studies, although there was no difference compared with placebo intervention [40•]. Future randomized controlled studies of subgroups of OSA patients are needed to evaluate all the various symptoms that may affect sleep apnea patients. A recent study found that OSA patients that predominantly have sleep arousals more often continue OA treatment than those with predominant desaturations, indicating that the arousal subgroup might experience more subjective advantages from OA treatment [52]. Many symptoms such as insomnia, daytime sleepiness, headaches, and restless legs may coexist with OSA [53–55] and require individualized treatment approaches.

In conclusion, the effect of OAs on daytime sleepiness is uncertain, particularly in snorers and those with mild to moderate OSA, i.e., the group of patients for whom this type of therapy is primarily recommended. It will be important for the future to study various symptomatic effects of OA treatment in relation to phenotype. Maybe there are also other unknown outcomes on sleep of OSA treatment, such as on the glymphatic system [56], that might have an impact on symptoms.

## Effects on Snoring

Patients’ interest usually focuses on reducing snoring in order to eliminate disturbances in family life or on journeys. Most studies have reported subjectively registered effects on snoring, although some objective measurements have also been made [39, 47]. Snoring is more effectively controlled by OA than by an intraoral placebo device, while CPAP is more effective than OA [57–59]. Persistent snoring during OA treatment has been related to insufficient apnea control and poor adherence with treatment [58, 60•].

In conclusion, fairly little research has focused on the treatment effects on snoring, probably because of difficulty measuring sounds and less evidence of snoring being related to longer term negative health outcomes [1]. From the patients’ perspective, more research on the psychosocial outcomes produced by the treatment of snoring would be of interest.

## Health Outcomes

The health effects of OSA treatments have mainly focused on the cardiovascular effects. Blood pressure is reduced by OAs and to a similar level as with CPAP [61•]. In addition, OA has been found to improve the nightly dips in blood pressure [62, 63]. One small, descriptive study reports a similar mortality rate between patients treated with OA, CPAP, and healthy controls compared with untreated severe OSA patients [64]. A few more studies of small samples report improved endothelial [65, 66] and cardiac function during OA treatment [67].

One reason for the similar effects on cardiovascular health produced by OAs compared with CPAP has been related to the increased preference for and adherence with OA [68]. The use of compliance monitors for OAs in larger studies is needed to determine more exactly the long-term health outcomes of OA compared with CPAP [69]. Important tasks for future research will be to study harder endpoint outcomes of OA treatment in comparison with CPAP.

## Side Effects

Most side-effects of OA treatment are temporary and disappear during the first few months of treatment. Examples of these side-effects include pain and salivation problems [70]. An unforeseen aspect of the risk of side-effects was visualized in a recent study that discovered more muscular pain in the initial part of OA treatment in patients on statin medication than in controls [71].

Tooth movements from the forces that are applied to the teeth constitute the main problem. OAs produce distally directed forces on the upper dentition and forwardly directed ones on the lower teeth. In the long run, there will be a dose-dependent shift in dental occlusion, with the lower teeth moving into a more anterior position, the upper ones moving distally and a loss of chewing contact areas in the posterior parts [72–74]. Almost all patients can be expected to develop a decreased overjet and overbite during the first 5 years [75], while around one third of the patients will experience more than 1 mm of change [76]. Bite changes will increase progressively as long as treatment continues [77•]. In a group of patients that were evaluated after 17 years, the median change in overjet was −1.1 mm, and the median change in overbite was −1.6 mm [78•]. The maximum overjet change in one of these patients was −5 mm, and the maximum overbite change was −4 mm in another patient. Younger and older subjects are affected by bite changes to a similar degree [38], although this depends on oral health and the length of the treatment. Interestingly, only a minority of patients notice these bite changes [79, 80], which indicates that they are less disturbing than expected.

In conclusion, bite changes during OA treatment are progressive in nature, and patients may at some time point be disturbed about the esthetics or problems biting or chewing. Most importantly, these bite changes will influence the mechanism of the device, since a forward shift of the lower teeth compared with the upper ones will result in a successively reduced degree of mandibular advancement. This puts the efficacy of the treatment at risk. Consequently, patients must be cared for in an individual way, since they will respond differently to mandibular repositioning during the night. It may be important for the future to study how often patients have to be followed up in order to assess bite changes in relation to the efficacy of the device and the importance of bite changes for oral health.

## Long-Term Outcomes

**Fig. 1:**
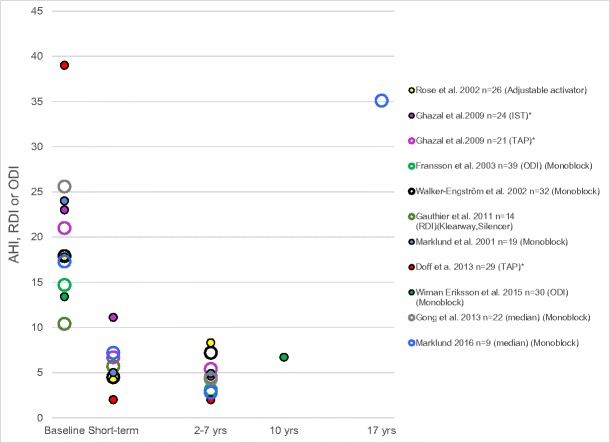
Efficacy in 10 long-term studies of OA treatment (the data refer to AHI, when not otherwise stated. * = baseline value from a larger sample)

The efficacy of OAs is fairly stable up to 10 years, according to nine small studies of carefully followed up patients [12, 70, 81–88] (Fig. 1). After that time, only one study has reported results from a few patients [78•]. In that study, all the patients increased their AHI with the device, and all but one patient increased the AHI without it. The weight of the patients did not increase, and they were not sleepy at the long-term follow-up. In addition, all OAs had been continuously adjusted with more advancement in order to compensate for the shift in dental occlusion, with the lower teeth successively being located more anteriorly in relation to the upper teeth as a common side-effect of the device. The deterioration in disease severity could have been explained by aging, which is related to an increase in AHI [89, 90]. It is important to be aware of the fact that the patients’ ability to keep their airways patent changes continuously. In addition, bite changes may reduce the actual advancement of the lower jaw and reduce the efficacy of the device. The mechanism of an OA is highly dependent on the pathophysiology of OSA and the actual advancement of the lower jaw. No study has yet presented the long-term effectiveness in a large group of patients treated with OAs for sleep apnea and evaluated which patients are primary long-term candidates for this kind of treatment and which patients require more intense surveillance.

In conclusion, the effectiveness of an OA will decrease in the longer term, since some patients discontinue treatment because of poor subjective effects or side-effects, and continuing patients may risk a poorer objective treatment effect [91]. It is not the same patient that is treated after a number of years, because of age and bite changes. The mechanism of action of OAs is more vulnerable than that of CPAP, which makes it necessary more continuously to re-evaluate the treatment outcome. Much more research is needed about the long-term outcomes of OA therapy. In addition, it would be of interest to study oral health during OSA treatment, i.e., whether oral health improves or deteriorates as a result of the treatment, and, in that case, in which patients, or whether both things occur in the majority.

## OA Design

OAs exists in many different designs, and few studies have dealt with the influence of various design details on the efficacy and side-effects of the treatment. The most common and effective [36] types of device, the titrable ones, are constructed into two parts. There is an upper part and a lower one, with an intermaxillary adjustment mechanism between them in order to allow the lower jaw to be moved forward successively and find an optimal mandibular position. The two parts may be fixed to one another, with a screw mechanism, for example, or the lower jaw may be allowed to move laterally and/or vertically. Studies have indicated that appliances that allows mouth opening are less effective in reducing sleep apnea [35, 92]. One of these studies tested, in a small sample, the influence of elastics to hold the jaws together in a specific device that allowed mouth opening in its original design [35]. Most patients experienced no difference in efficacy, while two patients with severe disease experienced a halving of apnea frequency in the supine position with the elastics in place compared with not having them. The use of non-individualized appliances cannot be recommended, based on the present level of knowledge [93, 94]. The retention of these non-individualized devices is often poorer than with a custom-made design [94], although this might be improved in the future [95].

Side-effects and patients’ experiences have been sparsely evaluated in relation to device design. One study reports that patients stop treatment because of uncomfortable appliances [96], a fact that is of great importance to treatment outcome.

The degree of bite changes is influenced by appliance design, in addition to the initial type of bite and treatment time. One study revealed no change in overjet and overbite after 4-year treatment [97]. That study used a specific OA design, with a lack of buccal coverage on the upper incisors and enforced lower incisor coverage. An observational study found fewer changes in overjet and overbite with a soft elastomeric device that covered some parts of the alveolar processes in addition to all the teeth compared with a hard acrylic OA with full occlusal coverage that was mainly fixed to the teeth [76]. A specific orthodontic oral appliance with incorporated forces to counteract the posteriorly directed forces on the upper front teeth had a positive effect on overjet changes compared with a control device in a small group of patients [98]. Most likely, a number of design details will influence the treatment outcome and side-effects in several ways.

In conclusion, there are numerous OA designs and a lack of identified golden standard types of device. Custom-made, titrable OAs that do not allow mouth opening are primarily recommended today. Prefabricated devices often have poor retention and are therefore not suitable as test devices either. Although much is already known about the outcome of OAs in the treatment of OSA, knowledge about various device designs is still lacking.

## Other Aspects of the Treatment

The recent article by McNicholas et al. [3] discusses indications for treatment in patients with milder disease. In the light of the large numbers of patients that are affected by the disorder and whether it is healthy always to eliminate sleep apneas in all patients, the more exact indications were discussed [99•].

The role of sleeping together has been elucidated in a recent review article [100]. Differences in diurnal preferences and Chrono types between individuals, genders, and cultural norms may affect daytime functioning and relationships. The article stresses that bed partners should be more directly involved in various treatment options for different sleep disorders, among which snoring and breathing pauses during sleep constitute an important part. It would be interesting to study adherence and motivation with sleep apnea treatments in relation to this topic in more detail [101].

## Conclusions

OAs reduce sleep apneas and are an effective treatment for selected patients.

Upper airway pathophysiology, such as pharyngeal collapsibility and verified widening by mandibular advancement, constitute promising predictors of success for the effects of OA therapy.

The effects on daytime sleepiness by OAs are uncertain and are influenced by OSA severity and other causes of sleepiness.

There are promising effects on cardiovascular health by OA therapy.

Bite changes occur in almost all patients, although they tend to be minor and do generally not disturb the patients.

The influence of device design on the efficacy and side-effects of OAs is almost unknown.

Long-term efficacy is good up to 10 years in well-controlled samples, but it probably declines in the real-life, long-term perspective. The natural development of the disease usually results in a deterioration in AHI. Effectiveness declines, with patients discontinuing treatment because of poor subjective treatment effect or side-effects, or poorer outcomes in follow-up sleep apnea recordings.

## Future Research

It is time to look at the outcomes of OAs in various phenotypes of obstructive sleep apnea patients.

More validation of predictors is needed.

More studies of the symptomatic effects of OSA treatments are needed, since they are still elusive.

Bite changes might influence the mechanism of the device, and methods to minimize these adverse effects are needed.

The many designs of OAs require comparison in terms of efficacy, stability, and side-effects.

The longer term outcomes in various areas, in particular cardiovascular health and mortality in real life, should be studied. The “Orange” project will shed light on the real-life outcomes of OA therapy, also in the longer term [102].
